# Study on Magnetization Roasting Kinetics of High-Iron and Low-Silicon Red Mud

**DOI:** 10.3390/ma16186178

**Published:** 2023-09-12

**Authors:** Lei Xie, Jiao Hao, Chaojie Hu, Hanquan Zhang

**Affiliations:** School of Resources & Safety Engineering, Wuhan Institute of Technology, Wuhan 430073, China; xieleizhuxiaohong@163.com (L.X.); hj13100690226@163.com (J.H.); 18155897381@163.com (C.H.)

**Keywords:** red mud, magnetization roasting, kinetics, mineral phase reconstruction

## Abstract

High-iron and low-silicon red mud is not only an alkaline solid waste from Bayer process alumina production, but it is also a very important secondary iron resource. Magnetization roasting is considered as an effective and typical method for the iron recovery and removal of impurities in red mud. In this work, based on the characteristics of large specific surface area and high porosity of red mud, the kinetics of magnetization roasting and phase transformation of red mud were studied. Thermodynamic analysis results show that the reduction of iron oxide in red mud is more easily promoted by CO as reducing agent at low roasting temperature. The reduction reaction is prone to overreduction, and fayalite and ferrospinel can be formed in the reaction system. The phase transformation and iron reduction mechanism during the roasting process were evaluated. Most of hematite and goethite in the red mud decomposed in the process of magnetization roasting, released CO_2_, and transformed into strongly magnetic magnetite. The reaction process has some characteristics controlled by homogeneous reaction. The process of magnetization roasting reduction with CO was controlled by the hybrid control dynamics model, and the apparent activation energy was 38.31 kJ·mol^−1^.

## 1. Introduction

Red mud is an aluminum industry byproduct and is generated by digestion of bauxite ore in caustic solution during the Bayer process [1]. Typically, around 0.9~1.6 tons of red mud are produced per ton of alumina produced. Every year, about 180 million tons of red mud are produced all over the world, and more than 5 billion tons of red mud had accumulated in reservoirs globally by 2021 [2,3,4,5,6]. About 100 million tons of red mud have been produced annually in China at present, mainly distributed in Shandong, Shanxi, Henan, and Guangxi provinces, accounting for 88% of the total [5,6,7]. Generally, red mud is stockpiled in open yards, causing serious problems such as soil, water, and air pollution, cultivated land occupation, and the destruction of ecological environments [8].

The iron content of red mud of the Bayer process is relatively high, mainly existing in the form of limonite and goethite. Moreover, red mud contains a lot of valuable metal elements, such as titanium, chromium, zirconium, niobium, scandium, and other rare metals and radioactive elements [9,10]. Moreover, red mud has the characteristics of fine particles, porosity, strong alkalinity, and radiation. At present, the resource recovery rate of red mud in the world is about 15% on average and only 4.5–6.0% in China. The comprehensive utilization of red mud includes two directions. One is to extract valuable components in red mud, such as the recovery of iron, aluminum, titanium, scandium, and other metal elements. The methods of extracting valuable metal from red mud are conventional physical methods, the pyrometallurgical method, and hydrometallurgical methods. Another method is to use red mud as a comprehensive mineral raw material, such as environmental protection functional materials, wall materials, cement, mine filling, and so on [11]. To realize the full quantitative comprehensive utilization of red mud, it is necessary to economically extract and enrich valuable elements in red mud [12]. For example, the iron in the red mud is recovered by the selective smelting technology, and scandium, uranium, and thorium in the red mud are recovered by the resin inhalation dissolution process [13,14]. The red mud slag was treated by the chlorination roasting process, and a high content of TiO_2_ was obtained; Al_2_O_3_ and V_2_O_5_ were also enriched in the slag.

In China, the external bauxite has a high iron and low silicon content, and the Bayer red mud produced is predominantly high-iron bauxite red mud, with the iron content close to 40%, and more than 50% of the iron minerals is alumogoethite with weak magnetics. Efficient recovery of iron from red mud not only has great economic value and environmental benefits but also helps to solve the problem of lack of iron ore resources in China. At present, numerous investigations have been carried out on the recovery of iron from high-iron red mud. The methods can be summarized as physical separation, reduction roasting–magnetic separation, and acid leaching, mainly including high-intensity magnetic separation, gravity separation, reduction smelting method, reduction roasting–magnetic separation method, and acid leaching [15]. The physical separation processes are simple, clean, and environmentally friendly, and their operational costs are low compared to other methods such as the pyrometallurgical process. However, the overall iron recovery is generally low, and the content of impurities is high with these physical separation methods. The acid-leaching method has the advantages of a high leaching rate and simultaneous leaching of multiple metals. However, due to the high alkalinity of RM, acid leaching requires a large amount of acid to neutralize the alkalinity in RM, which leads to the problems of high acid consumption and strong acidity of leaching residue.

Agrawal [16] compared the carbothermal reduction reactions using a muffle furnace and microwave heating, followed by magnetic separation to recover iron from Indian red mud. They found that microwave heating significantly improves the TFe and recovery rate of iron at a lower time and with less reducing agent. In a microwave furnace, they finally obtained iron concentrate assaying 47 wt% Fe with an iron recovery of 88% at optimal conditions of 1000 °C, 10 min with 11% charcoal. Microwave heating provides faster reduction, a cleaner process, and less energy and reductant consumption. Sadangi [17] investigated the effects of the amount of coal used, reduction temperature, reduction time, selection of pellet size, and grinding fineness on the iron recovery and grade. The results showed that magnetic concentrate containing 65.93% iron value with a recovery of 61.85% was obtained at optimal conditions of 1150 °C, 60 min with coal amount of 25%. The abovementioned red mud direct reduction technical route has a higher metallization rate and recovery rate, but lower yield. The reaction temperature is above 1000 °C, the equipment investment is large, and the energy consumption is high. The grade of iron concentrate powder is not more than 50%, the recovery rate is only 50~60%, and it can only be preselected. High-quality iron concentrates could be obtained by high-temperature reduction roasting, but the process is costly and consumes high energy. Low-temperature reduction roasting is of low energy consumption, low cost, relatively clean, and environmentally friendly, but the TFe grade of iron concentrate is relatively low.

In recent years, magnetization roasting–magnetic separation has been a promising method to process oxidized iron ore such as medium- and low-grade limonite and siderite. The magnetization roasting temperature is usually 600~700 °C, which is much lower than the direct reduction temperature (above 1000 °C), and has the advantages of being a simple process, low energy consumption, a high conversion rate, and environmental friendliness. Magnetic separation tailings have high pozzolanic activity and high quality for building materials [18]. It has a positive effect on the full quantitative and efficient utilization of secondary resources, such as medium- or low-iron-grade ore resources and sulfuric acid slag, and has been widely concerned. Yuan, S. and Sumedh [19,20,21,22,23,24] carried out the magnetization roasting–magnetic separation treatment of red mud. It was found that most hematite or goethite is converted to magnetite under the action of a reducing agent, and a small amount of newly formed magnetite is reoxidized to strong magnetic maghemite. Samouhos [25] studied the separation of iron oxide from red mud by hydrogen prereduction and magnetic separation under static conditions. By controlling roasting time, roasting temperature, and hydrogen concentration, the maximum conversion rate of hematite to magnetite reached 87% at 480 °C.

Magnetization roasting can effectively treat hematite, goethite, and other iron-bearing minerals in red mud and achieve the purpose of iron extraction and impurity reduction, by converting weakly magnetic hematite, limonite, or goethite to strongly magnetic magnetite, and using low magnetic separation to separate iron concentrate. When the overreduction phenomenon occurs, a small amount of iron exists in the form of spinel and ferro olivine, resulting in a high iron content in the tailings. During the roasting process, the activity of red mud changed significantly. Because the structural variation of gangue minerals such as aluminosilicate causes a large number of Si-O bonds and Al-O bonds to break, a large number of active particles are generated, and this shows good gelling performance, so the activity of the magnetized roasting and magnetic separation tailings is good. The dynamic model of magnetization roasting is different from the typical gas–solid unreacted nuclear shrinkage model, which makes the thermal control complicated. Because the red mud has fine granularity, high porosity, and strong diffusion and penetration ability of the reducing agent, it is therefore necessary to study the phase transformation and kinetic law in the magnetization reduction process of red mud, in order to provide theoretical support for the control of the magnetization roasting process of weakly magnetic iron ore with complex composition.

Considerable research has been conducted on the magnetization roasting reduction kinetics of conversion of natural hematite to magnetite, and its theoretical system is relatively well developed [26,27,28]. Gao [29] has shown that the conversion rate of hematite increases with the increase of magnetization roasting time and temperature, but decreases with the increase of particle size, indicating that particle size has a great influence on the roasting behavior of hematite, because the reduction reaction first occurs at the edge of the particle and gradually proceeds to the inside of the particle. The difference in the thickness of the new magnetite produced by the different particle size of hematite significantly affects the diffusion of the reduction gas, which is also the main reason for affecting the reduction roasting process.

According to the equilibrium relationship of iron oxides under different CO partial pressure and temperature conditions and the characteristics of red mud, it is very important to study the effect of hydrogen instead of carbon magnetization roasting reduction behavior and gas composition on the magnetization reduction kinetics. Ponomar [30] conducted the dynamic analysis of magnetization reduction of hematite. The result shows that the reduction of hematite to magnetite can be described by a first-order reaction model. The reaction rate decreased with the extension of exposure time. The reaction rate constant increases by more than 20 times with the increase in temperature, and the obtained kinetic model can estimate the energy cost of the conversion of low magnetic iron ore to magnetite relatively quickly. Because Bayer red mud has a high content of hematite and goethite, fine particle size, and large specific surface area, the control of reducing atmosphere and reducing process deserves more attention. Serious overreduction may be caused due to the low temperature and fast conversion rate during magnetization roasting. Therefore, it is important to study the reduction process of hematite or goethite in red mud to magnetite under different reduction conditions. It also reveals the dynamic law of rapid transformation in a weak atmosphere and determines the kinetic model and control link. It provides a theoretical basis for further optimizing the thermal system of magnetization reduction roasting.

## 2. Materials and Methods

### 2.1. Materials

The raw material selected in the experiment was Shandong red mud, and the solid reducing agent pulverized coal comes from Wulongquan Mine, and its properties are shown in Table 1. It can be seen that pulverized coal has a high fixed carbon content of 52.59% and ash content of 10.11%. The carbon monoxide content used in base roasting is 99.99%, and its concentration is changed by mixing nitrogen. HCl, H_2_SO_4_, H_3_PO_4_, K_2_Cr_2_O_7_, TiCl_3_, SnCl_2_·2H_2_O, and Na_2_WO_4_ were all analytically pure.

### 2.2. Instruments and Methods

#### 2.2.1. Roasting Test

Sample pretreatment: The sample with a grain size below 0.85 mm is mixed evenly with the reducing agent in a certain proportion. After dilution with 10% water, the powder sample was pressed to a 10 mm × 10 mm mass with an oil press at 1 t pressure.

**Figure 1:**
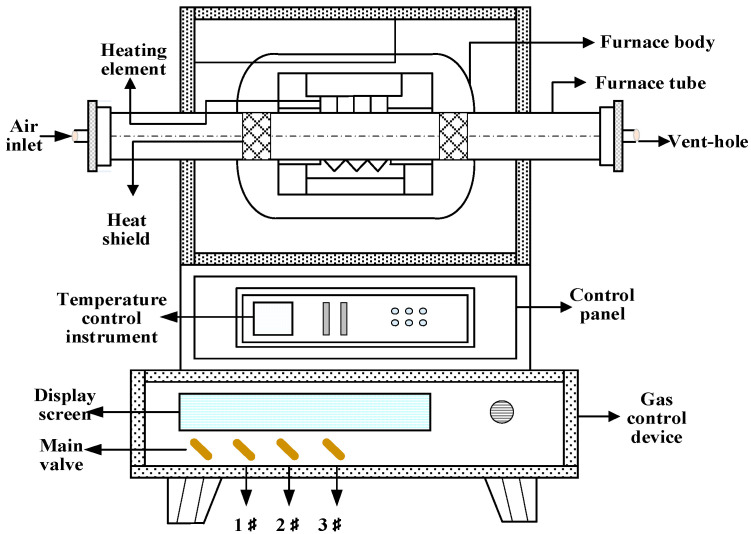
Magnetized roasting tube atmosphere furnace.

Tubular atmosphere furnace roasting: Arrange the pressed masses in a boat-type corundum crucible and place them in the middle of the furnace. Set the temperature, atmosphere concentration, and time of the atmosphere furnace, and the system begins to enter the preheating stage, slowly heating up, and the temperature range is 0–500 °C. Open the nitrogen valve and the main valve of the gas control device, and set the nitrogen flow rate, so that the furnace is filled with nitrogen and other gases are discharged during the preheating stage. When the temperature reaches the target temperature, the system enters the roasting stage, adjusts the ratio of nitrogen and CO, and passes the mixed gas into the furnace tube. The temperature remains constant until the end of the phase. After the end of roasting, the system temperature began to drop, and CO was stopped and N_2_ was continued. Ensure that oxygen does not enter the furnace during the cooling process to prevent oxidation of the product. When the temperature drops to the set temperature, turn off the main switch of the temperature control device, and then close all the gas valves when it is at room temperature. The reduction furnace used in the test study is a tubular atmosphere furnace, as shown in Figure 1.

Magnetic separation of roasting product: After the roasted product is broken to less than 1 mm, it is finely ground and sorted by a magnetic separator.

#### 2.2.2. Analytical Method

The determination of total iron and ferrous content is based on international standards and potassium dichromate volumetric analysis. The instrument used for X-ray diffraction (XRD) analysis is the D8 ADVANCE X-ray diffractometer in Bruker, Germany. The radiation source is a Cu target, the step width is 0.02°, and the scanning angle ranges from 10 to 70°. The X-ray fluorescence (XRF) was performed on an X-ray fluorescence spectrometer from PANalytical Axios in Panaco, the Netherlands. SEM observation was performed on the Ge8 miniSEM300 scanning electron microscope. The acceleration voltage of the instrument is 0.02 KV–30 KV, the resolution is 0.7 nm. Scanning electron microscopy can observe the morphology and structure of the surface of the material, and it can also analyze the surface elements. The thermogravimetric analysis test used a gallop thermogravimetric analyzer, and the samples were heated from 30 °C to 850 °C under a nitrogen atmosphere.

## 3. Results and Discussion

### 3.1. Characterization Results of Raw Materials

The red mud was a red, powdery block structure with a total iron content of 41.54%. The main chemical analysis and iron phase analysis of the samples using XRF are shown in Table 2. The highest content of red mud was iron oxide, which reached 60.91%, followed by Al_2_O_3_ and TiO_2_, which were 15.47% and 4.79%, respectively. The contents of SiO_2_ and CaO are only 3.82% and 1.29%, respectively, indicating that the red mud is of a high-iron, high-aluminum, and low-silicon type.

**Figure 2:**
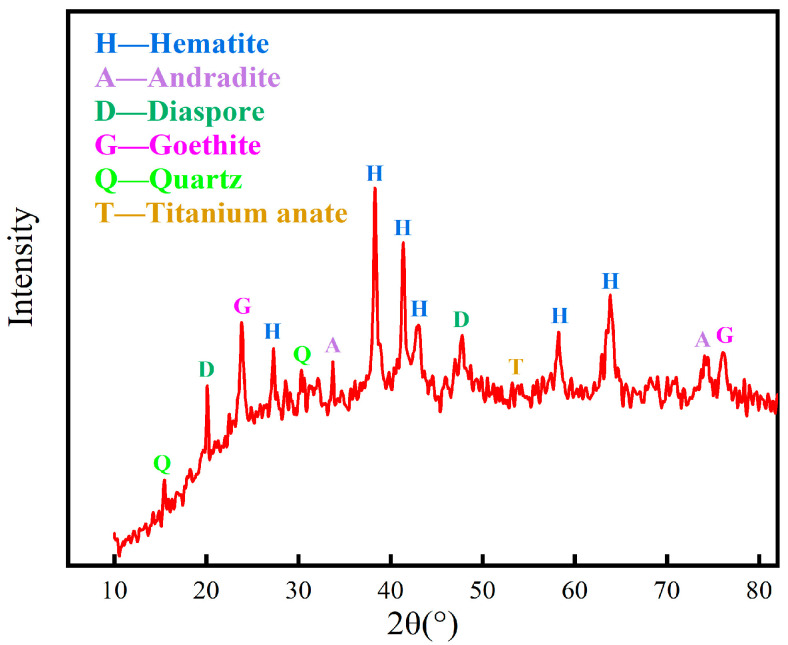
XRD spectrum of red mud.

The mineralogical composition was analyzed by the X-ray diffraction method, and the results in Figure 2 show that the minerals in the red mud are complex in composition. The main mineral phases were found to be hematite, andradite, goethite, anatase, and quartz. The different grain grades of red mud were analyzed, and the results are shown in Table 3. The particle size of red mud is fine, and the particle size less than 0.038 mm accounts for about 50%; the iron grade of this part of red mud is only 38.98%, while the iron grade of particles larger than 0.038 mm is more than 42%.

**Figure 3:**
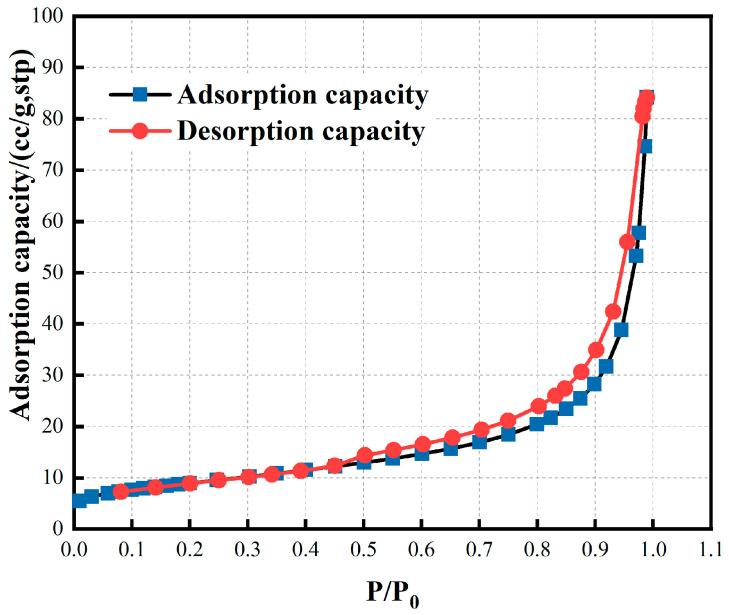
N_2_ adsorption–desorption isotherms.

The specific surface area of red mud is large, which is 100–200 times that of ordinary iron ore powder of the same particle size (1000–2000 cm^2^/g) (Table 4). The BET surface area of red mud is 32.18 m^2^/g. As shown in Figure 3, the nitrogen adsorption–desorption isotherms curves of the red mud belong to type III adsorption isotherm according to the Brunauer–Emmett–Teller (BET) classification. The slow increase of nitrogen uptake at low relative pressure (P/P_0_ < 0.5) implies transition from a single-molecular layer to a multimolecular layer, whereas the sharp increase and the hysteresis loop at high relative pressure (P/P_0_ = 0.5–1.0) indicate the presence of mesoporous and macroporous materials in the red mud. There was no adsorption saturation when the vapor pressure reached saturation. At this time, large-pore volume filling occurs due to capillary condensation, which makes the adsorption capacity increase rapidly.

**Figure 4:**
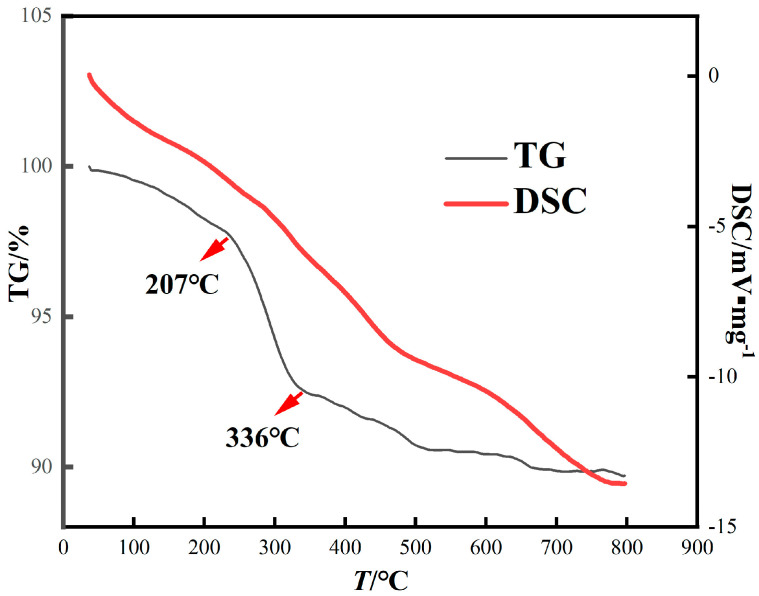
TG-DSC curve of red mud.

In order to explore the physical and chemical changes of red mud during roasting, thermogravimetric (TG) analysis of red mud was performed at 0~800 °C (Figure 4). The steep fall in curves from 0 °C to 800 °C indicates a strong interaction of CO with red mud, and the total mass loss was 10.29%. These curves are slightly different, which are due to the different chemical composition of the red mud. They mainly show similar curves, and the curves can be divided into three stages. In the first stage (36~207 °C), the weight loss of red mud is 1.85%, mainly due to the decomposition of part of the crystal water. In the second stage (207~336 °C), the weight loss is about 5.21%, and the weight loss rate is faster. At this point, the boehmite and goethite decompose into hematite [31]. In the third stage (336~559 °C), the red mud continued to lose weight by 2.35%, which may be related to the decomposition of calcite [32]. According to the DSC curve, the red mud is in an endothermic state; thus, a reducing agent must be added for the reaction to occur.

### 3.2. Magnetization Reduction Thermodynamics of Iron Oxide in Red Mud

Common minerals in red mud are diaspore, diaspore, rutile, goethite, hematite, calcite, calcite, and quartz, etc. Explore the magnetization reduction thermodynamics of hematite and interfering elements such as aluminum and silicon (goethite will decompose into original hematite structure at about 300~400 °C). When pulverized coal is used as a reducing agent, the reactions that may occur in red mud are as follows:

2Fe_2_O_3_(s) + C(s) → 4FeO(s) + CO_2_(g) (1)

6Fe_2_O_3_(s) + C(s) → 4Fe_3_O_4_(s) + CO_2_(g) (2)

2FeO(s) + C(s) → 2Fe(s) + CO_2_(g) (3)

FeO(s) + SiO_2_(s) → FeSiO_3_(s) (4)

FeSiO_3_(s) → FeO(s) + SiO_2_(s) (5)

2FeSiO_3_(s) + C(s) → 2Fe(s) + 2SiO_2_(s) + CO_2_(g) (6)

FeO(s) + Al_2_O_3_(s) → FeAl_2_O_4_(s) (7)

FeAl_2_O_4_(s) → FeO(s) + Al_2_O_3_(s) (8)

2FeAl_2_O_4_(s) + C(s) → 2Fe(s) + 2Al_2_O_3_(s) + CO_2_(g) (9)

**Figure 5:**
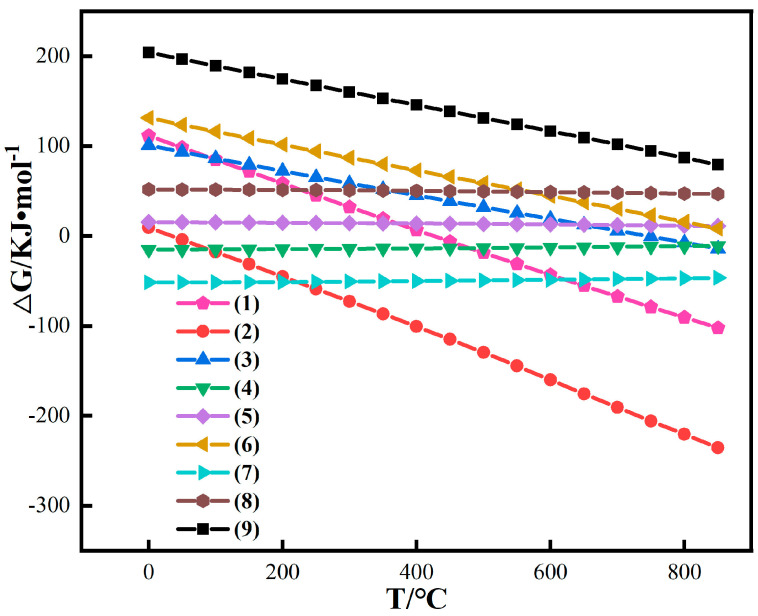
Thermodynamic calculation of simple iron oxides in red mud.

HSC-Chemistry 6.0 software was used to perform thermodynamic calculations of the above reactions, and the calculation results are shown in Figure 5.

When the reducing agent is CO, the main components of the red mud may react as follows:

Fe_2_O_3_(s) + CO(g) → 2FeO(s) + CO_2_(g) (10)

3Fe_2_O_3_(s) + CO(g) → 2Fe_3_O_4_(s) + CO_2_(g) (11)

FeO(s) + CO(g) → Fe(s) + CO_2_(g) (12)

FeSiO_3_(s) + CO(g) → Fe(s) + SiO_2_(s) + CO_2_(g) (13)

FeAl_2_O_4_(s) + CO(g) → Fe(s) + Al_2_O_3_(s) + CO_2_(g) (14)

**Figure 6:**
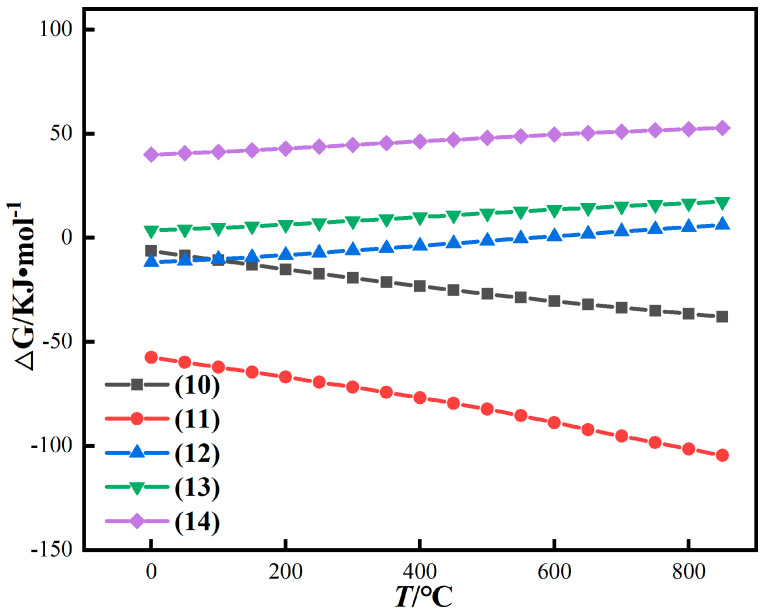
Thermodynamic calculation results of iron oxide reduction in red mud under gas-based conditions.

After thermodynamic calculation, the calculation results are shown in Figure 6.

Figure 6 shows that the conversion of Fe_2_O_3_ to Fe_3_O_4_ is easier than that of Fe_2_O_3_ to FeO when CO is used as a reducing agent. Different from coal-based conditions, the reaction of conversion of Fe_2_O_3_ to Fe_3_O_4_ and FeO can always be spontaneous under gas-based conditions in the temperature range of 0~850 °C. However, under gas-based conditions, the change trend of Gibbs free energy of reaction is slower than that of coal-based reaction with the increase in temperature. Therefore, the reduction reaction under CO atmosphere at low temperature is easier than that with pulverized coal [33,34,35]. The Gibbs free energy of reactions (13) and (14) is positively correlated with temperature, and ∆G is always greater than 0, inferring that CO has difficulty in reducing iron olivine and iron spinel alone at any temperature.

### 3.3. Study on Iron Reduction Kinetics of Red Mud during Magnetization Roasting

According to the author’s previous research results, the optimum conditions of coal-based red mud magnetization roasting are as follows: roasting temperature is 700 °C, roasting time is 50 min, the amount of pulverized coal is 8%, the mass proportion of grinding particle size less than 0.045 mm is 60%, and the magnetic field intensity is 68.8 KA/m. The grade of magnetic concentrate is 56.08%, and the recovery rate is 87.89%.

The best conditions for magnetization roasting of red mud under gas-based conditions are as follows: roasting temperature is 650 °C, roasting time is 30 min, CO concentration is 20%, the mass proportion of grinding particle size less than 0.045 mm is 80%, and the magnetic field intensity is 68.8 KA/m. The grade of iron concentrate is 57.19% with an iron recovery of 91.25%. Compared with coal-based reduction, gas-based reduction has a lower roasting temperature and shorter roasting time. After magnetic separation, the grade and recovery of magnetic separation iron concentrate are improved slightly.

At present, there are three methods to study the kinetics of magnetization roasting. The first method is to use the CO and CO_2_ content in the tail gas to calculate the pyrolysis kinetics of the iron phase. The second method is to calculate the dynamic model of red mud by measuring the change of FeO content in roasted ore with time. The third method is to calculate the roasting kinetics by measuring the change in quality of the roasted ore with time under the condition of constant temperature or temperature rise. The second method is used to calculate the kinetic model of the iron phase in red mud, and the reduction degree is calculated by testing the ferrous content in the roasted ore, so as to obtain the relationship between the reduction degree and the roasting time.

In the reduction process of red mud, the external diffusion resistance has little effect on the boundary of red mud clumps, which is difficult to be a limiting factor in the reduction reaction of red mud. The reduction roasting process of red mud is mainly controlled by the internal diffusion of the mass and its interfacial chemical reaction. According to the current research, the factors that limit the reaction rate of magnetization reduction roasting in red mud may be interfacial chemical reaction, carbon gasification reaction, internal diffusion, and a hybrid control dynamics model [36].

(1) Interfacial chemical reaction [37]

The stoichiometric expression of the reduction reaction is shown as follows:

(15) $$\underset{B}{\sum }v_{B}=0$$

When the reaction rate is controlled by the interfacial reaction, the chemical reaction rate can be expressed as:

(16) $$v_{c}=-\frac{dn_{A}}{dt}=4k\pi r_{i}^{2}C_{Ai}$$

According to Equation (16), the kinetic equation can be derived as:

(17) $$t=a\left[1-{\left(1-x\right)}^{1/3}\right]$$

where *x* represents reduction fraction, *α* represents the degree of reduction: *α* = (FeO/TFe) × 100%, *k* represents the reaction rate constant. The activation energy of the interface reaction control model ranges from 40 to 300 kJ/mol. Equation (17) is deformed to obtain Equation (18).

(18) $$1-1-\alpha^{\frac{1}{3}}=kt$$

(2) Carbon gasification reaction [38]

The relationship between reduction degree α and reaction rate constant *k* is as follows:

(19) $$In\left(1-\alpha \right)=-kt$$

(3) Internal diffusion [39]

When the reaction is controlled by the diffusion of gas within the pellet, according to the diffusion law, the reaction rate *r_d_* of the gas in the solid-phase product layer can be expressed as follows:

(20) $$r_{d}=-\frac{dn_{A}}{dt}=4\pi r_{0}^{2}d_{A}\frac{d_{cA}}{d_{r}}$$

*α* represents the reduction degree, *k* represents the reaction rate constant, and Formula (21) is derived.

(21) $$1-\frac{2}{3\alpha }-1-\alpha^{\frac{2}{3}}=kt$$

(4) Hybrid control dynamics model

The mixed control kinetics model is influenced by both internal diffusion and chemical reaction control effects, where the roasting reaction rate is as follows:

(22) $${\left(1-x\right)}^{-\frac{1}{3}}-1+\frac{1}{3}\mathrm{In}\left(1-x\right)=kt$$

**Figure 7:**
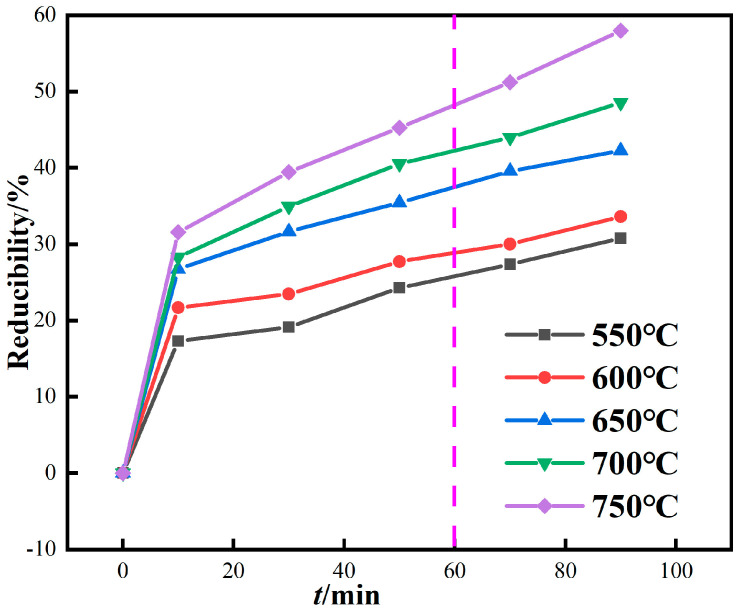
Relationship between reduction degree and time of red mud by magnetization roasting based on gas.

When the CO concentration is 20% and the roasting temperature is 600 °C, 650 °C, 700 °C, 750 °C, and 800 °C, the influence of roasting time on the reduction degree of iron in red mud is studied, and the results are shown in Figure 7. In magnetization roasting, the theoretical reduction degree of hematite or goethite is 42.8%. As shown in Figure 7, when the roasting temperature is above 650 °C and the reduction time exceeds 60 min, the reduction degree exceeds the theoretical value of magnetization roasting, and there is an obvious overreduction phenomenon.

**Figure 8:**
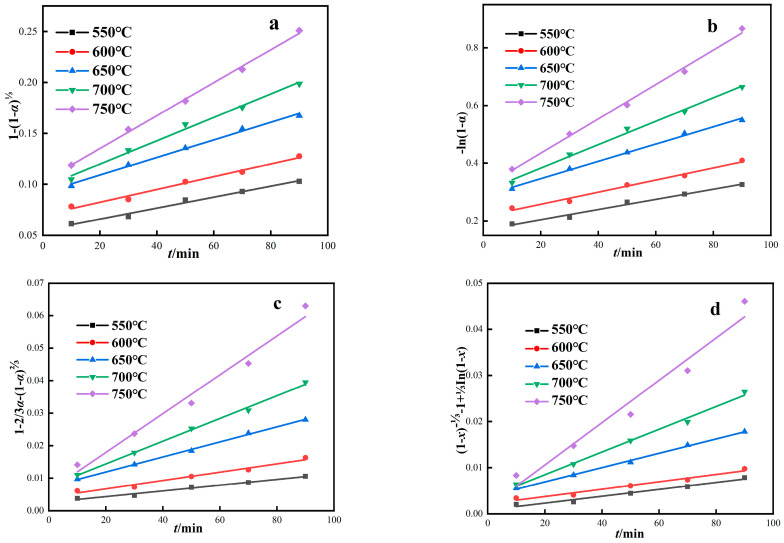
Kinetic model of red mud magnetization roasting under gas-based conditions. ((**a**) Interface reaction control model, (**b**) Carbon gasification reaction control model, (**c**) Internal diffusion control model, (**d**) Hybrid control dynamics model).

According to four dynamic control models, the roasting test results were linearly fitted under different thermodynamic temperatures. As shown in Figure 8 and Table 5, reaction rate constants (k_1_, k_2_, k_3_, k_4_) and correlation coefficients (R^2^) of the four kinetic models were obtained from linear equation parameters. The correlation coefficient reflects the matching degree of different control models to the magnetization roasting process. The red mud magnetization reduction roasting process fits the four control models well, and the correlation coefficients of the four models are all above 0.95. From the fitting results, it is impossible to determine which control model is more consistent with the magnetization reduction roasting process of red mud, and further study is needed.

The Arrhenius equation is used to calculate the activation energy. The control model of the roasting process is further judged by verifying whether the activation energy conforms to the apparent activation energy range of the kinetic control model.

The Arrhenius theorem is: $k=Ae^{-\frac{E_{a}}{RT}}$. By calculating the apparent activation energy of the reaction, the integral equation of the Arrhenius rate formula can be obtained:

(23) $$Ink=-\frac{E_{a}}{RT}+InA$$

where:

*k* is the reaction rate constant;

*E_a_* is the activation energy (kJ·mol^−1^);

*T* is the thermodynamic temperature (K);

*R* is the perfect gas constant (8.314 J·mol^−1^·K^−1^);

*A* is the frequency factor;

*InA* is an integral constant.

**Figure 9:**
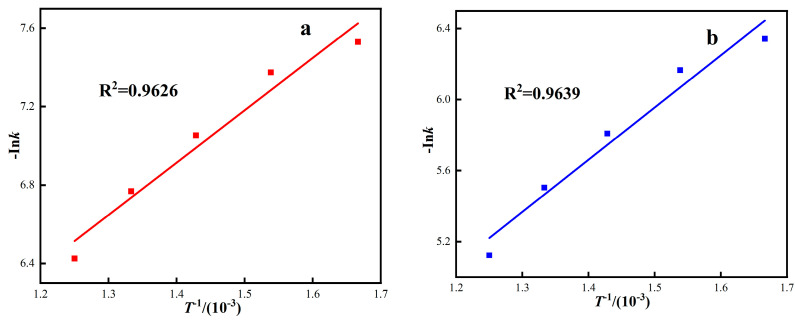

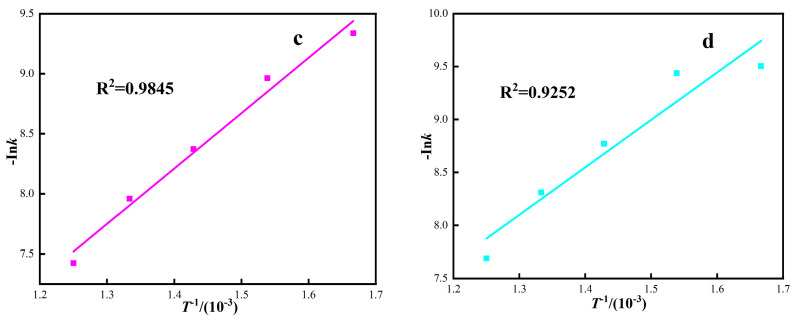
The relationship between Ink and 1/T under different response control models. ((**a**) Interface reaction control model, (**b**) Carbon gasification reaction control model, (**c**) Internal diffusion control model, (**d**) Hybrid control dynamics model).

At different thermodynamic temperatures, 1/T was plotted by *Ink* according to four dynamic control models, and the corresponding fitting curve was obtained, as shown in Figure 9. The apparent activation energy of the reaction was calculated by fitting the curve equation parameters, and the calculation results are shown in Table 6. As can be seen from Figure 9 and Table 6, the four dynamic control models are well fitted, and the correlation coefficients R^2^ are all above 0.90. The activation energies of the interfacial chemical reaction control model, carbon gasification reaction control model, internal diffusion control model, and hybrid control dynamics model are 22.18 kJ·mol^−1^, 24.47 kJ·mol^−1^, 38.31kJ·mol^−1^, and 37.25 kJ·mol^−1^, respectively.

Under gas-based conditions, the correlation coefficient R^2^ of the red mud diffusion control model is higher, and the fitting activation energy is 38.31 kJ·mol^−1^. The apparent activation energy represents the difficulty of a chemical reaction and can be used to judge the limiting link of reaction rate. According to the relationship between the apparent activation energy of iron oxide reduction and the rate control link, as shown in Table 7. The rate control link of this roasting process is controlled by the diffusion in the gas and the interfacial chemical reaction [40,41,42]. Because the red mud has the characteristics of small particle size, high porosity, and large specific surface area, during the roasting process, the mass transfer resistance is relatively small, which has some characteristics that the homogeneous phase should be controlled, and the diffusion resistance of the reducing medium has little influence.

### 3.4. Iron Phase Transformation of Red Mud during Magnetization Roasting

**Figure 10:**
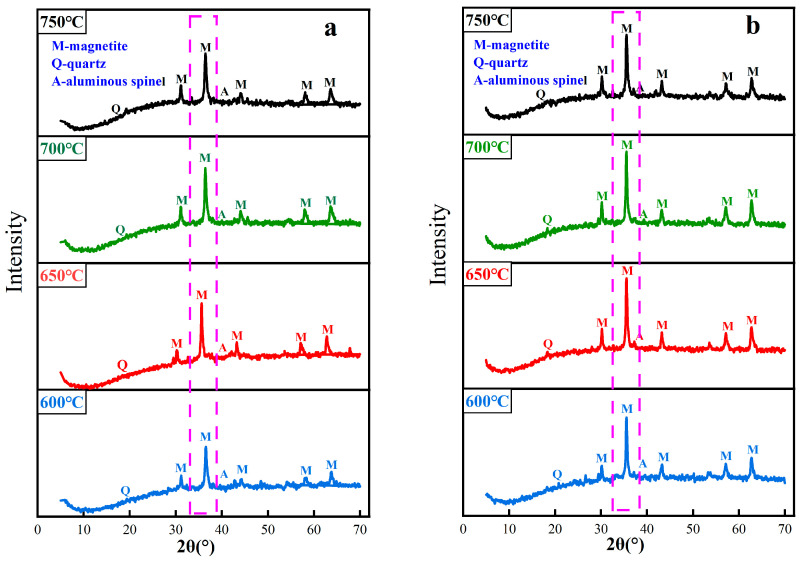
XRD patterns of magnetized calcined ore at different temperatures. ((**a**) Coal-based magnetization roasting, (**b**) Gas-based magnetization roasting).

When the roasting temperature is in the range of 600~750 °C, the phase analysis of coal-based and gas-based roasted ores is carried out, and the results are shown in Figure 10. After magnetization roasting, hematite and goethite in the red mud are transformed into magnetite, and some quartz and a small amount of aluminum spinel exist. Figure 10a shows that during coal-based magnetization roasting, the diffraction peak intensity of magnetite increases with the increase in temperature. When the roasting temperature is 700 °C, the intensity of the diffraction peak is the highest, and the intensity of the diffraction peak begins to decline as the temperature continues to rise. As shown in Figure 10b, during gas-based magnetization roasting, the diffraction peak intensity of magnetite is the highest when the roasting temperature is 650 °C. When the temperature continues to rise to 700 °C, the change in diffraction peak intensity is not obvious, and when the temperature rises to 750 °C, there is a downward trend. By comparing the phase transformation rules of the two kinds of roasted ore, it is found that the temperature of gas-based magnetization roasting is lower than that of coal-based, and the diffraction peak intensity of magnetite is obviously higher than that of coal-based.

## 4. Conclusions

The present study adopted an approach to utilize abundantly generated red mud as a source of iron values. The reduction kinetics and iron phase transformation of iron during the magnetization roasting of red mud with CO were investigated. The main conclusions are as follows:

(1) The iron minerals in red mud are mainly limonite and alumogoethite, with a total iron content of 41.54%. The particle size of red mud is fine, and the specific surface area of red mud is 100–200 times larger than ordinary iron ore powder of the same particle size. The BET surface area of red mud is 32.18 m^2^/g.
(5) According to the results of thermodynamic analysis, CO is easier to promote the reduction of iron oxide than coal at low reduction temperature as a reducing agent. In the process of magnetization roasting, there will be iron olivine and iron spinel in the reaction system, which are difficult to be decomposed by pulverized coal or CO reduction.
(6) The study of the magnetization roasting control model shows that the magnetization roasting process of red mud with CO conforms to the hybrid control dynamics model. The activation energy is 38.31kJ·mol^−1^, which is different from the typical diffusion control model. There is an overreduction phenomenon in the reduction reaction. The control model of the magnetization roasting process is different from the typical unreacted nuclear shrinkage model, and the reduction process has some characteristics of homogeneous reaction control.
(7) The phase transformation law shows that hematite and goethite in the red mud are transformed into magnetite after magnetization roasting. In addition, there is some quartz and a small amount of aluminum spinel in the red mud. The optimum temperature of gas-based magnetization roasting is 650 °C, and that of coal-based magnetization roasting is 700 °C. The effect of gas-based magnetization roasting is better than that of coal-based magnetization roasting.

## Figures and Tables

**Table 1:** Industrial analysis of pulverized coal (wt%).

Moisture	Volatile Matter	Ash	Fixed Carbon	Sulfur
7.7	29.07	10.11	52.59	0.64

**Table 2:** Major chemical composition of the red mud (wt%).

Compositions	Fe_2_O_3_	TiO_2_	SiO_2_	Al_2_O_3_	CaO	MgO	Na_2_O	K_2_O	SO_3_	Cr_2_O_3_	P_2_O_5_	MnO
content	60.91	4.79	3.82	15.47	1.29	0.20	2.85	0.04	0.13	0.17	0.19	0.08

**Table 3:** Red mud particle size analysis.

Grain Size/mm	Proportion/%	TFe/%	Iron Distribution/%
+0.15	4.45	45.15	4.71
−0.15~+0.075	21.98	48.09	24.80
−0.075~+0.045	19.98	44.87	21.04
−0.045~+0.038	4.58	42.91	4.61
−0.038	49.01	38.98	44.83
total	100.00	42.61	100.00

**Table 4:** Surface characteristics of the red mud determined from N_2_ adsorption–desorption isotherms.

Sample	S_BET_ (m^2^/g) ^α^	S_mic_ (m^2^/g) ^β^	S_ext_ (m^2^/g) ^γ^
The raw material of red mud	32.18	2.59	29.59

^α^ S_BET_ represents BET surface area; ^β^ S_mic_ was determined by the *t*-plot method; ^γ^ S_ext_ was obtained by subtracting S_mic_ from SSA.

**Table 5:** Kinetic model parameters of red mud magnetization roasting.

Temperature/°C	R^2^			
	Interface Reaction Control Model	Carbon Gasification Reaction Control Model	Internal Diffusion Control Model	Hybrid Control Dynamics Model
550	0.9864	0.9867	0.9862	0.9735
600	0.9860	0.9853	0.9751	0.9713
650	0.9945	0.9958	0.9983	0.9973
700	0.9917	0.9939	0.9966	0.9939
750	0.9975	0.9955	0.9799	0.9528

**Table 6:** Arrhenius model fits parameters.

Kinetic Models	*k*	R^2^	*E_a_*/kJ·mol^−1^
Interface reaction control model	2.6678	0.9626	22.18
Carbon gasification reaction control model	2.9395	0.9639	24.47
Internal diffusion control model	4.6087	0.9845	38.31
Hybrid control dynamics model	4.48	0.9252	37.25

**Table 7:** Relationship of rate-controlling step and activation energy of iron oxide.

*E*/kJ·mol^−1^	Rate-Controlling Step
8~16	Gas diffusion control
29~42	Internal diffusion and interfacial chemical reaction are controlled together
60~67	Control of interfacial chemical reactions
>90	Solid-phase diffusion control
